# Chimeric antigen receptor natural killer cells: a promising antitumor immunotherapy

**DOI:** 10.1002/mco2.422

**Published:** 2023-12-01

**Authors:** Yan Wang, Shengjie Jin, Qiqi Zhuang, Na Liu, Ruyi Chen, Sofia Abdulkadir Adam, Jie Jin, Jie Sun

**Affiliations:** ^1^ Department of Hematology The First Affiliated Hospital Zhejiang University School of Medicine Hangzhou Zhejiang China; ^2^ Key Laboratory of Hematologic Malignancies Diagnosis, and Treatment Hangzhou Zhejiang China; ^3^ Department of Oncology Affiliated Hospital of Weifang Medical University School of Clinical Medicine Weifang Medical University Weifang Shandong China; ^4^ Zhejiang University Cancer Center Hangzhou Zhejiang China; ^5^ Zhejiang Provincial Clinical Research Center for Hematological Disorders Hangzhou Zhejiang China

**Keywords:** cancer, chimeric antigen receptor, hematologic malignancies, immunotherapy, leukemia, NK cell, tumor microenvironment

## Abstract

Chimeric antigen receptor (CAR) T cells have been successfully used in adoptive cell therapy for malignancies. However, some obstacles, including side effects such as graft‐versus‐host disease and cytokine release syndrome, therapy resistance, limited sources, as well as high cost, limited the application of CAR T cells. Recently, CAR natural killer (NK) cells have been pursued as the effector cells for adoptive immunotherapy for their attractive merits of strong intrinsic antitumor activity and relatively mild side effects. Additionally, CAR NK cells can be available from various sources and do not require strict human leukocyte antigen matching, which suggests them as promising “off‐the‐shelf” products for clinical application. Although the use of CAR NK cells is restrained by the limited proliferation and impaired efficiency within the immunosuppressive tumor microenvironment, further investigation in optimizing CAR structure and combination therapies will overcome these challenges. This review will summarize the advancement of CAR NK cells, CAR NK cell manufacture, the clinical outcomes of CAR NK therapy, the challenges in the field, and prospective solutions. Besides, we will discuss the emerging application of other immune cells for CAR engineering. Collectively, this comprehensive review will provide a valuable and informative summary of current progress and evaluate challenges and future opportunities of CAR NK cells in tumor treatment.

## Graphical abstract

NK cells for CAR NK generation can be derived from several sources, including PBMC, iPSC, UCB, and NK‐92 cells. NK cells are transduced with CAR structure by viral or nonviral vectors after isolation or differentiation from a variety of sources, then CAR‐NK cells can be sorted. CAR NK cells are then cocultured with cytokines or feeder cells expressing IL‐15 and mb‐IL‐21 to allow further expansion of NK cells. This review will discuss the advances of CAR NK cells, the design improvement of CAR, CAR NK cell manufacture, the challenges, and the clinical outcomes in CAR NK cell therapy.

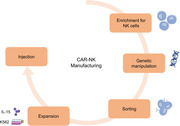

## 1 INTRODUCTION

The development of immunotherapy has revolutionized the field of cancer therapy. Instead of targeting the tumor cells directly, immunotherapy focuses on modifying immune effector cells or overcoming immunosuppression induced by tumor cells and the local microenvironment, endowing the immune cells with enhanced effect to target and eradicate malignant cells. Immunotherapies, including adoptive cell transfer (ACT) of genetically modified immune cells and inhibitors of immune checkpoints such as cytotoxic T Lymphocyte antigen 4 (CTLA‐4) and programmed death‐1 (PD‐1), have been applied in oncological clinical practice and obtained durable clinical responses.^1^

Earlier ACT, which directly infused activated autologous or allogeneic immune cells into cancer patients to eliminate tumor cells, had only achieved modest benefits in clinical trials.^2^ Therefore, the strategy that engineering immune effector cells, such as T cells, natural killer (NK) cells, iNKT cells, and macrophages, to express chimeric antigen receptors (CARs) has been developed for better recognizing and killing the tumor cells.^3^ CAR T cell therapy is showing impressive results in treating malignant hematological diseases and solid tumors. The complete remission rate reached up to 80% in CD19‐positive B cell acute lymphoblastic leukemia (B‐ALL) patients treated with CD19 CAR T.^4^ However, accompanying adverse effects such as severe cytokine release syndrome (CRS), neurotoxicity, and graft‐versus‐host disease (GVHD) have restricted the clinical application of CAR T therapy.^5^ Moreover, accumulating reports showed that some patients relapsed after CAR T cell therapy, which is associated with target antigen loss.^6^ These limitations aroused further exploration of other immune effector cells as candidates for CAR carriers.

NK cells are lymphocytes of the innate immune system, which eliminate cancer cells and infected cells through secretion of perforin and granzyme or death receptor‐mediated cytotoxicity with no need for prior activation.^7^ The expression of variable ligand‐binding receptors on the surface of NK cells enables correct identification and proper responses to different signals. The natural cytotoxicity receptors, such as natural cytotoxicity triggering receptor 30 (NKp30), NKp44, and NKp46, are the major activating receptors to initiate the lysis of the targeted cells.^8^, ^9^ The inhibitory receptors, such as inhibitory killer immunoglobulin‐like receptors (KIRs) and NKG2A, recognize major histocompatibility complex (MHC) class I molecules, and transmit inhibitory signals to prevent NK cells from overactivating.^10^ In recent years, NK cell has gained attention as an alternative to T cell in the field of immune cell engineering. Studies on CAR NK cells developed rapidly, which major focused on optimizing the CAR structure to overcome current challenges, such as limited efficacy and persistence of CAR NK cells in the tumor microenvironment (TME). At the same time, encouraging clinical trial results of CAR NK products paved the way for more widespread use of CAR NK cells in clinical practice.

In this review, we described the detailed structure and manufacturing process of CAR NK cells, and how CAR NK cells are activated. We also discussed current challenges and evolving strategies to promote persistence and killing capacity against tumor cells. Finally, we provided a comprehensive overview of the application of CAR NK cells for the treatment of solid tumors and hematologic malignancies.

## 2 THE CHALLENGES IN CAR T THERAPY AND THE ADVANTAGES OF CAR NK THERAPY

Hitherto, barriers such as severe side effects, unsustainability, and high time and financial consumption still limit the broad adoption of CAR T cell therapy. On the contrary, the NK cell has the potential to become an outstanding option for CAR therapy because of its intrinsic cytotoxicity, high efficacy and controllable adverse effects. The comparison of CAR T and CAR NK therapy is summarized in Table 1.

**TABLE 1:** The comparison of CAR NK and CAR T therapy.

	CAR NK	CAR T
Cell sources	Autologous and allogeneic PBMC, HSC, iPSC, UCB, NK‐92 cells^11^	Autologous or HLA‐matched allogeneic T cells^12^
Cost and time for manufacture	Less expensive and more accessible due to the “off‐the‐shelf” product	Costly and labor intensive due to personalized treatment^11^
CAR transduction	Difficult, especially the frozen and naive cells	Easier modification^13^
Activation	CAR dependent, ADCC, “missing‐self” recognition	CAR dependent^11^
HLA restriction	No	Yes^13^
GVHD risk	Barely appear	Early development of skin reactions and liver injury^14^
CRS and neurotoxicity	Barely appear	Severe but manageable^14^
Ongoing and completed clinical trials	More clinical trials are ongoing or recruiting	Efficacy and safety have been demonstrated in multicenter clinical trials

Abbreviations: ADCC, antibody‐dependent cellular cytotoxicity; HSC, hematopoietic stem cells; iPSC, induced pluripotent stem cells; PBMC, peripheral blood mononuclear cell; UCB, umbilical cord blood.

Activation and expansion of CAR T cells lead to massive serum cytokines accumulation, which contributes to the potential risks of CRS and neurotoxicity.^15^ Patients who suffered from CRS typically show a series of symptoms, including hypotension, fever, and respiratory failure.^16^ Neurotoxicity is another adverse event of CAR T therapy, which manifests in patients as seizures and phrenitis.^17^ Compared with CAR T cells, CAR‐NK cells are less likely to induce CRS and neurotoxicity due to a shorter lifespan and a cytokine secretion spectrum different from that of T cells.^18^ During CAR T cell therapy, macrophages are activated and secrete high levels of proinflammatory cytokines such as IL‐6 and IL‐1, triggering CRS and severe neurotoxicity.^19^ However, NK cells mainly produce interferon‐γ (IFN‐γ) and granulocyte‐macrophage colony‐stimulating factor (GM‐CSF), which are less likely to induce the CRS. Additionally, the production of IFN‐γ by NK cells is generally transient.^18^, ^19^, ^20^ Moreover, allogeneic T cells can cause GVHD, even after HLA matching.^21^ NK cells, do not induce GVHD, making them become optimal sources for a broad generation of safe off‐the‐shelf therapy products.^22^ In fact, the infused MHC‐mismatched NK cells can reduce GVHD severity by inhibiting T cell activation while retaining the graft‐versus‐tumor effect.^23^

Although impressive clinical efficacy against malignant diseases has been achieved, relapse still occurs commonly in patients after CAR T therapy.^24^ Loss of target antigens is one of the reasons for CAR T therapy failure.^25^ On the one hand, target antigens such as CD19 can be mutated by tumor cells under selective pressure, making antigens unrecognizable to CAR T cells, which is the most common mechanism of antigen loss.^26^ On the other hand, through a process called trogocytosis, antigens on tumor cells are removed by CAR and transferred to T cells, which decreases the target density of tumor cells.^27^ Furthermore, T cells separated from the PB of patients for CAR engineering are inevitably contaminated by a number of tumor cells, which can be transfected with the CAR genes, leading to an epitope masking by the CAR itself.^28^ The CAR‐expressing tumor cells persist throughout the manufacturing process and are finally infused back into patient.^29^ However, CAR NK cells can be activated and eradicate tumor cells through CAR‐dependent and CAR‐independent pathways. Apart from identifying the antigen on target cells through single‐chain fragment variable fragment (scFv), the surface activating receptor of NK cells can also bind to ligands on the target cells to exhibit cytotoxicity toward tumor cells.^30^ In addition, the antitumor activity of CAR NK cells can be redirected by IgG antibodies. For example, CD16a (FcγRIII), a receptor expressed on NK cells, can recognize fragment crystallizable region (Fc) of IgG and mediate ADCC.^31^ Moreover, to evade recognition by T cells, tumor cells downregulate the expression of MHC I molecules, whereas NK cells can identify and kill tumor cells lacking self‐MHC class I molecules via “missing‐self” recognition.^32^ Consequently, CAR‐NK cells exhibit better elimination efficacy while loss of antigens occurs in target cells.

T cells for CAR T cell generation are collected and separated from the patient's blood cells, which require complicated manufacturing processes, making CAR T therapy an expensive and time–cost treatment,^22^ which serves as another obstacle to the wide application of CAR T therapy. However, NK cells for CAR NK production can be from various sources, including autologous or allogeneic PB, UCB, HSC, iPSCs, and the NK‐92 cell line, which makes NK cell therapy a potential off‐the‐shelf cell therapy product.^9^ Therefore, the cost and preparation time for CAR NK cells can be reduced significantly, which could greatly benefit patients.^33^

## 3 CAR DESIGN OF CAR NK CELLS

**FIGURE 1:**
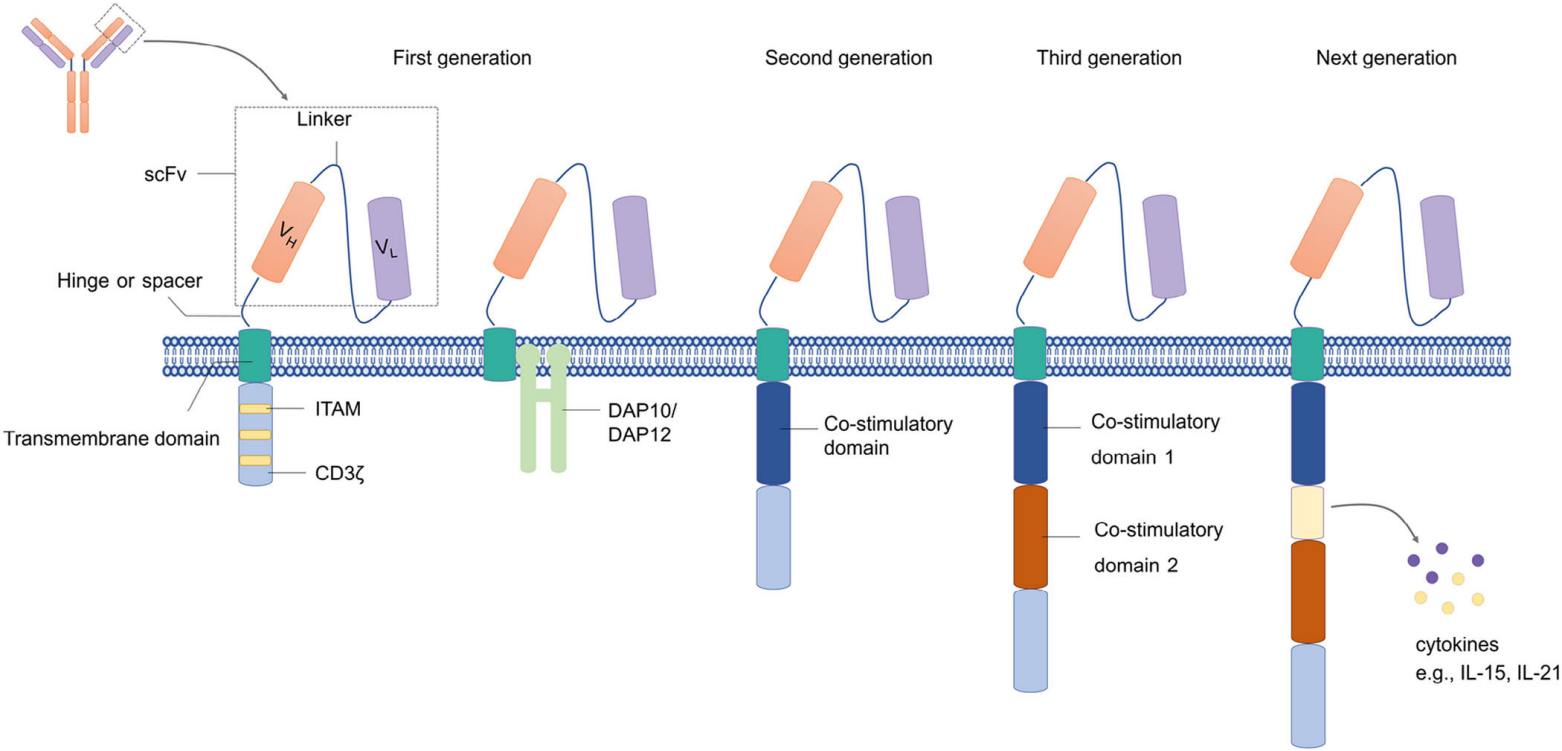
Development of the CAR structure. The structure of NK‐CAR consists of an extracellular portion, a TM domain, and an intracellular domain. The extracellular domain typically derives from the variable light (VL) and variable heavy (VH) regions of an antibody (scFv). The hinge links the scFv to the transmembrane domain. The TM domain is often derived from CD28 or CD8. The CAR design has been developed into the fourth generation. The intracellular domain of first‐generation CARs contains a fragment derived from CD3ζ but lacks a costimulatory domain. While the second and third‐generation CARs have one and two costimulatory domains respectively, besides the CD3ζ fragment. CARs containing DAP10 or DAP12 intracellular domain intracellular domain have also been developed, which is the adaptor protein associating with activating receptors of NK cells, and transmits activation signaling. The next‐generation CARs contain an additional protein fragment such as cytokines or inducible suicide proteins.

The CARs expressed in NK cells and T cells share similar structures, which usually consist of an extracellular portion, a transmembrane (TM) domain, and an intracellular domain (Figure 1). The extracellular domain typically comprises a scFv, which is derived from a tumor‐specific antibody and recognizes the target antigen, a linker that connects the heavy chain and light chain, and a hinge (also known as spacer) connecting the scFv to the TM region.^34^ The TM domain connects the extracellular region of the CAR to the intracellular domains, which is mainly derived from CD3ζ, CD8, or CD28.^35^ The intracellular region contains a signaling domain generally derived from the CD3ζ molecule. With the development of CAR manufacture, the costimulatory domain has also been applied in the intracellular domains, which improved the activation and persistence of CAR T cells.^36^, ^37^ The first‐generation CAR T cells showed a limited capacity for persistence and proliferation in vivo as they lacked the costimulatory signaling required for full activation.^38^ Therefore, the second‐generation CARs with a costimulatory domain and the third‐generation CARs with two costimulatory domains derived from CD28 or 4‐1BB were created to improve the immune response to target cells.^39^ Compared with the first‐generation CARs, the latter two are more popular owing to the increased level of expansion, persistence, and infiltration into tumor tissues.^40^ However, no evidence suggests that the third‐generation of CARs is superior in improving the primary response rate compared with the second‐generation CARs.^41^

### 3.1 Extracellular domain

The order of Fv, length of linker, and type of linker of the scFV did not noticeably affect the expression level of CAR on T cells. However, the surface expression of CAR can be improved by grafting complementarity‐determining region to the murine‐derived stable framework region in Fv, demonstrating that CAR expression efficiency and stability could be affected by the Fv structure.^42^

### 3.2 Hinge and TM region

The hinge region is the extracellular structural region of the CAR connecting the scFv units to the TM domain.^35^ Generally, this unit is derived from CD4, CD8α, CD28, and 4‐1BB. Increasing evidence showed that the function of CAR T cells was influenced by the type and length of the hinge. CAR T cells with short extracellular spacers showed a superior cytotoxic effect toward target cells compared with the ones with longer spacers, and inappropriate length of spacers may lead to off‐target effects.^41^, ^43^ In addition, optimizing the length of the spacer may facilitate the formation of optimal immunologic synapse and specificity of CAR NK cells via adjusting the distance between effector cells and target cells.^41^ The TM domain spans the cell membrane and connects the extracellular domain of the CAR to the intracellular signaling domains. Zhao et al.^44^ compared glypican‐3 (GPC3) CARs with different TM structures from 4‐1BB or CD8α, and the results demonstrated that CARs with 4‐1BB TM domain expressed at a higher level on the surface of T or NK cells compared with CARs with CD8α TM domain even with similar transfection efficiency. Furthermore, T and NK cells with 4‐1 BB TM domain exhibited superior cytotoxicity and a higher level of IFN‐γ secretion.^44^ The efficacy of CAR with 4‐1BB TM was only evaluated against a cell line of HCC ex vivo, and further experiments in vivo are needed to validate the killing effect against other targets. On the other hand, CARs with hinge and TM regions from either CD8α or CD28 exhibited similar abilities to decrease tumor burden in mice, but CAR T cells with CD8α derived hinge and TM domains may be safer and persist for a longer time in vivo due to decreased cytokines and lower levels of activation‐induced cell death.^45^

### 3.3 Intracellular signaling domain

The first‐generation CARs contain only one activating signal, CD3ζ, while the second‐ and third‐generation CARs contain one and two costimulatory domains, respectively.^46^ Most costimulatory molecules are derived from the tumor necrosis factor receptor family, including 4‐1BB, OX40, CD40, and CD27, and the immunoglobulin superfamily like CD28 and ICOS.^47^

Although extensive activation signals are necessary to induce a strong antitumor response, the effector cells could also be committed to a rapid exhaustion state. Therefore, the combination of costimulatory domains is important to regulate the response of adaptive immune cells. Compared with CD28/CD3ζ‐based CAR structure, 4‐1 BB/CD3ζ‐signaling induced slower and reduced downstream protein phosphorylation. However, the CD28/CD3ζ CAR T cells with more quick and intense phosphorylation signaling exhibited a less potent antitumor effect in mice models despite higher levels of immune cytokine production early after CAR ligation. In contrast, the weaker CD3ζ immunoreceptor tyrosine‐based activation motif (ITAM)‐signals in 4‐1 BB‐based CAR T cells exhibited better antitumor effect and longer persistence.^48^ Moreover, 4‐1BB‐CD3ζ signals induce the generation of sustainable, long‐lasting protective memory against tumors.^49^

In almost all the CAR NK cells, the CD3ζ transmits signals based on the interaction between CAR and target antigens.^50^ However, Katrin Töpfer et al. found that DNAX‐activation protein 12 (DAP12), an adapter protein associating with several activating receptors of NK cells, including NKG2C and NKp44,^51^ based CAR showed superior antitumor capacity compared with NK cells expressing a CD3ζ‐based CAR both in vitro and in vivo.^52^

### 3.4 Activation of CAR NK cells

**FIGURE 2:**
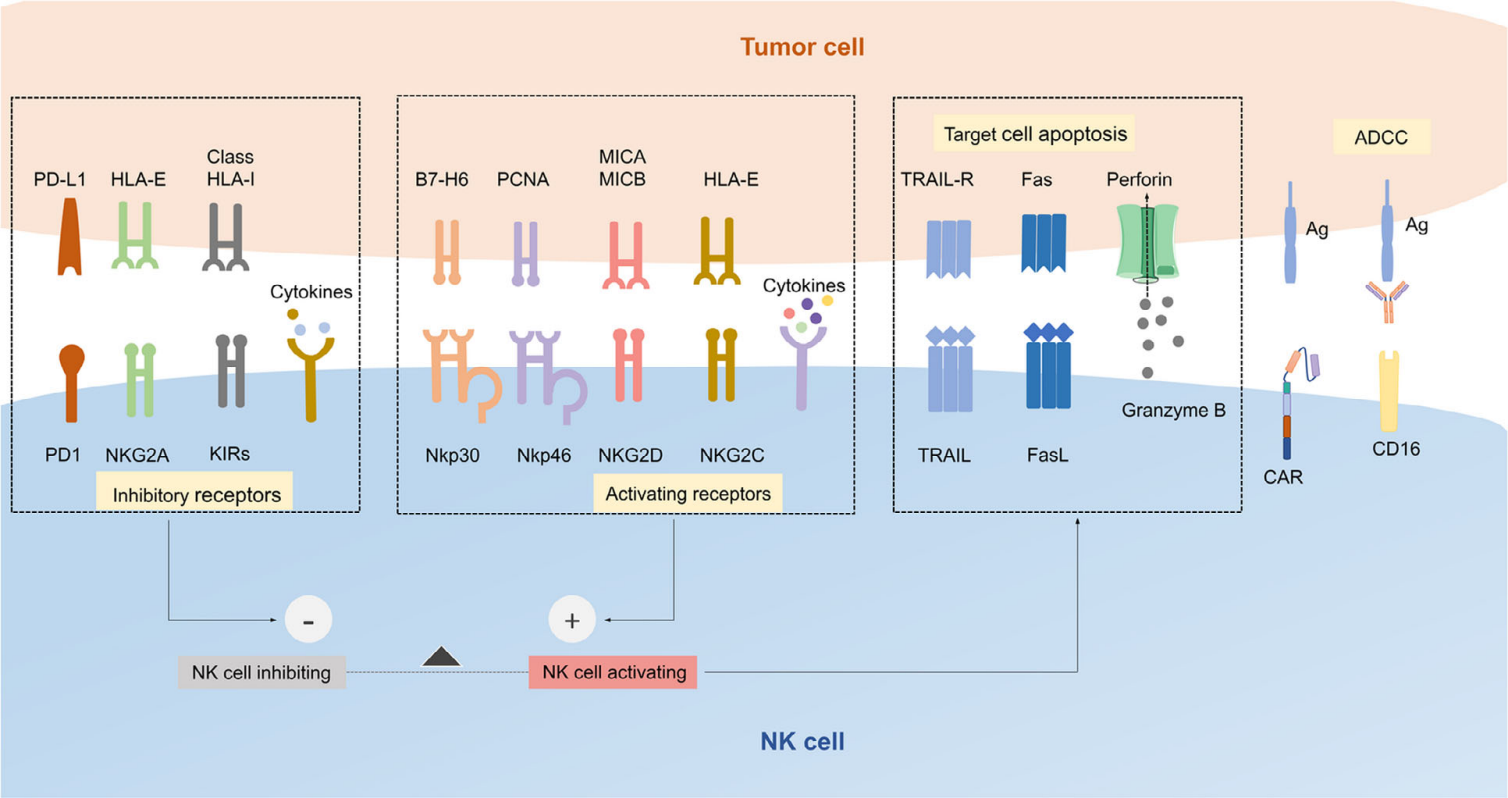
NK cell activation. The activation of NK cells is the result of the integrated signaling of intrinsic activating and inhibitory receptors, providing NK cells with the ability to rapidly eliminate target cells via death receptors, granzyme, and perforin. IgG targeting on tumor cells engages the CD16 receptor on NK cells and induces ADCC. CAR expression endows the NK cells with the capacity to activate and eradicate target cells through antibody–antigen interactions.

Activation of CAR NK cells can be initiated through the recognition of specific target antigens on tumor cells by CAR expressed on NK cells, which transduce the activating signaling to the intracellular domains of the CAR, such as CD3ζ, DAP10, DAP12, and costimulatory domains, resulting in the downstream processes.^53^ In addition, the integration and balance of various signaling from inhibitory receptors, activating receptors, and cytokine receptors provide NK cells with the ability to recognize and rapidly eliminate target cells (Figure 2).^54^

#### 3.4.1 Intrinsic activation signaling

Activation of CAR NK cells depends on the integration signaling of intrinsic activating receptors, inhibitory receptors, and cytokine receptors, most of which share the common adaptor molecules and signaling pathways.^54^ Upon activation, NK cells are able to exert killing capacity quickly and effectively toward malignant or infected cells without prior sensitization. The various open chromatin loci encoding NK cell killing effectors, such as IFN‐γ endow it with the ability to rapidly exert effector capacity.^55^ Natural cytotoxicity receptors such as NKp30 and NKp44 recognize the tumor antigens and recruit ITAM‐containing adaptor proteins, which are phosphorylated by SRC family kinase, resulting in recruitment and activation of zeta chain‐associated protein kinase 70 (ZAP70) and spleen tyrosine kinase (SYK). Signaling molecules downstream, such as phosphatidylinositol 3‐kinase (PI3K), will be further phosphorylated and activated. Finally, degranulation of cytotoxic granules such as perforin and granzymes is induced by Ca^2+^ flux and cytoskeletal reorganization, enabling NK cells to lysis target cells.^56^ As another essential activating receptor, NKG2D transduces activating signaling via binding to the adaptor protein DAP10.^57^ NK cells can also activate ADCC via the binding of CD16 and the Fc portion of IgG antibodies on tumor cells.^58^ What is more, NK cells can be activated by cytokines such as IL‐2, IL‐7, IL‐12, IL‐15, and TNF‐αvia the Janus kinases (JAK) and signal transducer and transcription activating signaling pathways.^59^

Inhibitory receptors of NK cells, including KIRs, CD94–NKG2A heterodimer, LAG‐3, and TIM‐3, avoid unrestrained cytotoxicity and protect healthy cells from being attacked via engaging MHC‐I molecules.^8^ KIRs recognize the HLA‐C molecule, and NKG2A recognizes the expression of HLA‐E.^60^ The intracellular portion of both receptors contains two immunoreceptor tyrosine‐based inhibitory motifs, which recruit phosphatases SHP‐1, bind the N‐terminal SH2 domain, and activate the catalytic domain of SHP‐1.^61^ Activated SHP‐1 mediates the dephosphorylation of multiple signaling molecules, including Vav‐1, which is significant for Ca^2+^ flux and synapse formation, and finally inhibits NK cell activation.^62^

#### 3.4.2 Engineered activation signaling

CAR expression endows the NK cells with the capacity to capture specific target cells through antibody–antigen interactions. The most of costimulatory and signaling domains of CAR molecules applied for NK cells are similar to those for T cells. The latest CAR structures depend on the CD3ζ chain signaling domain, which contains three ITAMs, facilitating recruitment and activation of Syk or ZAP70 tyrosine kinases, or PI3K signaling to mediate NK cell activation.^63^ On the other hand, NK‐derived adapter molecules, such as DAP10, DAP12, and 2B4 were applied as costimulatory domains in CAR.^64^ DAP12 is expressed on the membrane of NK cells and is associated with activating receptors through complementary charged TM domains. Similar to other ITAM‐based adaptor proteins, tyrosines in the DAP12 are phosphorylated of the by Src family kinases when DAP12‐associated receptors bind to the ligand and transmit activating signaling via recruitment and activation of Syk or ZAP70.^65^, ^66^ Although DAP10 lacks ITAM, it can directly recruit a Grb2‐Vav1 intermediate and p58 PI3K to initiate the downstream tyrosine phosphorylation and mediate activating signaling for the activating receptor NKG2D on NK cells. 2B4 is an activating receptor from the signaling lymphocytic activation molecule family and carries an immunoreceptor tyrosine‐based switch motif in its cytoplasmic domain, which recruits and binds SLAM‐associated protein to transmit activation signals upon binding to its ligand CD48.^67^ As a coreceptor of NK cells, 2B4 requires the coactivation of other activating receptors, such as NKD2D and NCRs, to stimulate the cytotoxicity and cytokine secretion of NK cells.^68^

#### 3.4.3 Target cell killing

Once NK cells are activated, a synapse linking the NK cell with the target cell is formed, and cytotoxic granules such as perforin and granzymes transport toward the synapse, which will fuse with the plasma membrane and finally relased.^69^ Perforin forms the pores on the membrane of target cells and facilitates the entry of granzymes, which induce the apoptosis of target cells via cleaving various substrates such as Bid and caspase‐3.^53^ In addition, once stimulated by the cytokines such as TNF family proteins, TM molecules Fas ligand (FasL) and TNF‐related apoptosis‐inducing ligand (TRAIL) are expressed on the NK cells, which respectively bind to the Fas receptor or death receptors 4 and 5 on target cells and therefore trigger assembly of the death‐inducing signaling complex (DISC).^70^ Caspases 8 and 10 in target cells are activated by DISC and further stimulate caspase 3, 6, and 7, which finally result in cell apoptosis.^71^

Apart from direct cytotoxicity, activated NK cells could trigger and recruit other immune effector cells, including dendritic cells and macrophages, into tumor tissue via secreting various cytokines and growth factors, including CC‐chemokine ligand 5, IFN‐γ, TNF and IL‐13.^72^

## 4 CAR NK CELL MANUFACTURE

### 4.1 Sources of NK cells for CAR engineering

**FIGURE 3:**
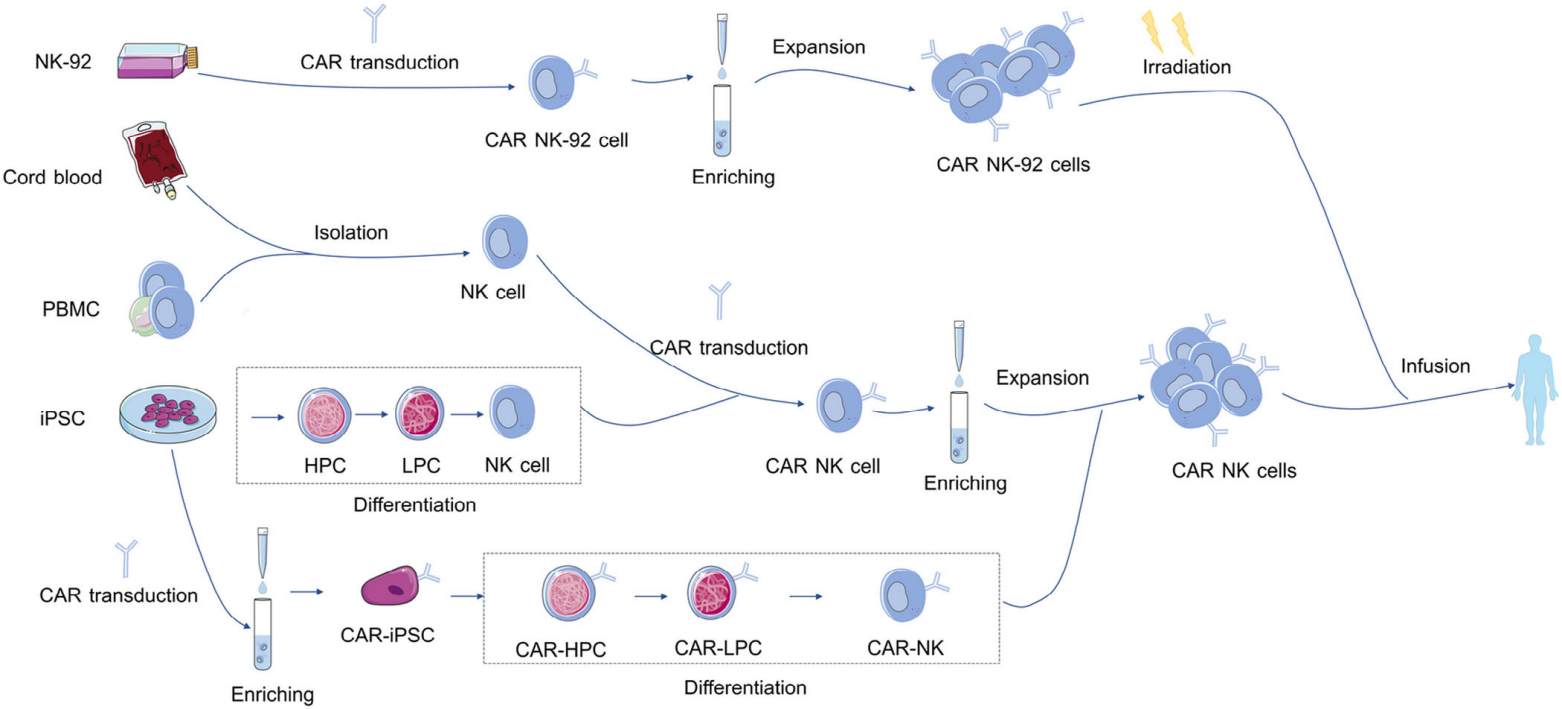
The process of CAR NK cell manufacture. NK cells for CAR NK generation can be derived from several sources, including PBMC, iPSC, UCB, and NK‐92 cells. NK cells are transduced with CAR structure typically by viral or nonviral vectors after isolation or differentiation from a variety of sources, then CAR‐NK cells can be enriched through magnetic‐activated cell sorting (MACS) or fluorescence‐activated cell sorting (FACS). MACS is more commonly used and showed higher purification rates compared with FACS. CAR NK cells are then cocultured with cytokines or feeder cells expressing IL‐15 and mb‐IL‐21 to allow further expansion of NK cells.

NK cells can be isolated and obtained from multiple sources (Figure 3). While allogeneic NK cells do not require strict HLA matching, the transplantation of HLA‐nonidentical NK cells could be possibly rejected by the host via MHC recognition.^73^ However, lymphodepletion chemotherapy before transplantation, including fludarabine and cyclophosphamide, is regularly administered to eliminate lymphoid cells of hosts and avoid graft rejection.^74^ Besides, HLA‐mismatch favors allogeneic NK cell‐mediated graft‐versus‐leukemia response in the absence of GVHD.^75^ Herein, we will discuss the upsides and downsides of NK cells from different sources for CAR manufacture (Table 2).

**TABLE 2:** Sources of NK cells for CAR engineering.

Sources	Advantages	Disadvantages
Peripheral blood	Mature phenotype^76^; strong cytotoxicity^77^	Account for only about 10% of the PB lymphocytes; autologous NK cells cannot kill target cells via “missing‐self” recognition^78^
Cord blood (CB)	Readily available from CB bank^79^; account for up to 30% of CB lymphocytes^80^	Highly express inhibitory receptor NKG2A^81^; low expression of granzyme B^82^
iPSC	High expansion capacity; easy to modify and engineer^83^	Complicated differentiation protocol^84^; low ADCC due to low CD16 expression^85^
NK‐92	Unlimited source; easily transfected and manipulated; less sensitive to freezing and thawing process^86^	Lack of ADCC due to absent CD16 expression; need irradiation before infusion; limited persistence after irradiation^41^

#### 4.1.1 Peripheral blood‐derived NK cells

NK cells for CAR‐NK cell manufacture can be directly enriched from the PBMC.^87^ With a wide range of activating receptors expressed on the cell surface, PBMC‐derived NK cells are more favorable for CAR NK generation.^88^ Besides, NK cells separated from PB with mature phenotype are not necessary to undergo a complicated differentiation protocol, like iPSC‐derived NK cells.^35^ Nevertheless, NK cells account for only 10% of the lymphocytes in PB and thus need to be expanded further in vitro to generate a sufficient quantity for clinical application.^41^ In addition, autologous NK cells possess impaired cytotoxicity against tumor cells compared with allogeneic NK cells due to silenced function when encountering antigens that express self‐MHC.^35^

#### 4.1.2 CB ‐derived NK cells

CB NK cells can be available from large global CB banks and expanded further in the absence of cytokines.^79^ Moreover, NK cells account for less than 10% of the lymphocytes in PB but account for up to 30% of all lymphocytes in CB, which makes it easier to enrich NK cells from CB.^89^ Therefore, CB serves as an off‐the‐self source of NK cells for adoptive immunotherapy. The efficiency of CB‐derived CAR NK cells has been proven in solid tumors and blood cancers.^20^, ^33^, ^90^, ^91^ Liu et al.^20^ compared the efficacy of CAR NK cells from CB and PB of CLL patients. Results showed that CB‐derived NK cells performed a superior capacity in killing the Raji and CLL cells. Furthermore, relative to CAR NK cells from PB, CB CAR NK cells showed increased polarization of the microtubule‐organizing center, which is a necessary step of NK cell‐mediated cytotoxicity.^20^ However, CB‐derived NK cells are immunologically immature, characterized mainly by expressing higher levels of inhibitory receptors such as NKG2A and KIRs, which impaired killing activity in response to target cells.^41^

#### 4.1.3 iPSC‐derived NK cells

iPSCs can be obtained easily from various sources such as skin and PB.^92^ iPSCs can expand persistently in vitro keeping pluripotency and therefore serve as an alternative resource to produce a sufficient quantity of NK cells for adoptive therapy.^93^ In the presence of specific cytokines, CAR‐transduced iPSCs can differentiate into NK cells.^94^ Similar to NK cells from other sources, iPSC‐derived NK cells exhibit killing capacity against tumor cells through the secretion of cytolytic enzymes, secretion of proinflammatory cytokines, such as perforins, granzymes, IFN‐γ and TNFα, or direct cell contact‐mediated apoptosis through TRAIL and Fas–FasL interaction.^95^ Li et al.^96^ compared the effect of PB‐NK cells, iPSC‐NK cells, and mesothelin CAR iPSC‐NK cells in an ovarian cancer mice model. The result showed mesothelin CAR iPSC‐NK cells exhibited the best antitumor activity and led to the longest overall survival of tumor‐bearing mice.^96^ However, it takes 3−5 weeks for the differentiation of iPSCs to CAR‐NK cells, which is a time‐consuming process relative to directly collecting NK cells from PB.^35^

#### 4.1.4 NK‐92 cells

NK‐92 is an IL‐2‐dependent cell line derived from a patient with non‐Hodgkin's lymphoma and is widely used to generate CAR NK cells.^97^ Compared with PB‐derived NK, NK‐92 cells are easier to proliferate and transduce CAR genes. Moreover, NK‐92 cells do not include T cells, which may cause GvHD after infusion.^41^ However, it must be irradiated before being infused into recipient patients to avoid secondary tumorigenesis, resulting in reduced CAR NK cell duration in vivo.^86^ Repeated infusions may improve the persistence of NK‐92 cells in vivo, but this may trigger the immune response to antigens expressed in NK‐92 cells.^98^ Moreover, NK‐92 cells cannot mediate ADCC because of the lack of surface receptor CD16a.^99^ Therefore, NK‐92 cells expressing the high‐affinity CD16/FcγRIIIA were established to harness the cytotoxicity capability. The safety of CAR NK‐92 cells had been demonstrated in clinical trials even at high intravenous infusion.^100^ In addition, several studies have evaluated the efficacy of CAR NK‐92 cells.^101^ For example, Michael et al. constructed CAR NK‐92 cells targeting CD123, a surface antigen highly expressed on the surface of acute myeloid leukemia (AML) cells, by using a retroviral vector containing third‐generation CAR elements. Results indicated that CAR NK‐92 cells showed more significant anti‐AML activity than unmodified NK‐92 cells in vivo.^102^

### 4.2 Gene transfer for CAR expression

#### 4.2.1 Lentiviral and retroviral transduction

Lentiviral and retroviral vectors are commonly used to deliver CAR genes into NK cells based on their ability to integrate CAR genes into the host genome, resulting in highly efficient and stable expression of the CAR genes.^103^ It is reported that transduction efficiency through retroviral is higher than that through lentiviral in NK cells. Furthermore, NK cells with CD19 CAR delivered by retroviral vector showed more significant killing capacity as compared with NK cells with CD19 CAR delivered by the lentiviral vector. In addition, the application of vectofusin‐1 or retronectin is found to be beneficial for improving gene delivery. Nevertheless, viral‐mediated CAR gene transfection may cause oncogenic transformation and subsequent uncontrolled adverse events.^104^ It was reported that patients who received treatment with retrovirus‐transduced CD34^+^ cells suffered leukemia due to insertional mutagenesis.^105^

#### 4.2.2 Transposon

The transposon system transfers genes through a bi‐component vector system that consists of a transposon containing a gene flanked by inverted terminal repeats and a gene‐coding transposase. Transposase protein binds to the transposon ends of the cargo vector specifically, excising the CAR gene and integrating it into the genome of NK cells.^106^ Currently, two transposon systems have been used to generate CAR NK cells, including the sleeping beauty transposon and the piggyBac transposon.^107^, ^108^ Moreover, as a nonviral system, transposon‐based CAR transduction is safer and more cost effective than the viral‐based system, which requires more equipment and reagents.^108^

#### 4.2.3 Electroporation

Unlike the two methods introduced above, electroporation transduces the CAR gene into NK cells transiently and cannot integrate it into the genome DNA of NK cells, which may therefore avoid severe cytokine storm resulting from the permanent CAR expression.^35^ For example, Xiao et al.^109^ constructed NKG2D CAR NK cells through an mRNA electroporation approach to achieve a transient but efficacious expression of CAR. Three patients who suffered from metastatic colorectal cancer were infused with NKG2D CAR NK cells locally, and the results showed that NKG2D CAR NK cells reduced tumor burden effectively without persistent or severe adverse events.^109^ Moreover, optimizing the electroporation parameters makes it possible to efficiently transduce CAR structure up to 12 kb into NK cells.^110^

## 5 CURRENT CHALLENGES AND OPTIMIZATION FOR CAR NK THERAPY

### 5.1 Limited expansion

Expanding NK cells, especially PB NK cells, to satisfy clinical use in vitro is challenging.^8^ Adding cytokines, including IL‐2, IL‐12, IL‐18, and IL‐21, into the culture medium facilitates the proliferation and activation of NK cells.^35^ IL‐15 was proven to stimulate NK cell expansion and activation.^111^ Several IL‐15‐based methods have been developed for NK cell expansion. Incubation with additional IL‐12, IL‐15, and IL‐18 is an attractive option to induce memory‐like NK cells, which express high‐affinity IL‐2 receptors, promoting the proliferation of NK cells when exposed to exogenous IL‐2.^112^ In addition, coculture with the leukemia cell line K562 overexpressing 4‐1BB‐L and membrane‐bound IL‐15 (mIL‐15), which is a type of IL‐15 expressed on the surface of feeder cells, is an alternative way to acquire a large number of NK cells.^84^ K562 cells expressing mIL‐15 exhibited an enhanced ability to stimulate NK cells and recruit NK cells into tumor tissues.^113^ Moreover, coculture with 4‐1BB‐L expressing K562 feeder cells can significantly enhance the transduction efficiency of CAR genes. Compared with freshly‐isolated NK cells, the transduction rate increased 4 fold in NK cells when cocultured with K562 cells.^35^ On the other hand, NK cells cocultured with K562 with 4‐1BB‐L and mIL‐15, induced senescence after dramatic expansion of NK cells. Treatment of mIL‐21, significantly extended telomere and sustained ex vivo NK cell proliferation. Therefore, the combination of IL‐15 or 4‐1BB‐L with mbIL‐21 may be a promising strategy to obtain enough cells for clinical use.^114^ Another novel feeder cell system applying mIL‐21 expressing human B‐lymphoblastoid cell line 721.221 (221‐mIL‐21) was also evaluated. NK cells from PBMC and CB cultured with 221‐mIL‐21 feeder cells showed superior expanding capacity, purity, and antitumor activity compared with NK cells expanded with K562‐mIL‐21 feeder cells.^115^ The interaction of OX40–OX40L between NK cells and the feeder cells is crucial for NK cell expansion. NK cells were cultured with K562 cells, which were genetically engineered to express OX40L, and exposed to IL‐21 transiently. NK cells cultured with unmodified K562 cells expanded 303fold compared with 2000‐fold expansion using the innovative strategy in 4 weeks.^116^

### 5.2 Short life span

Another major limitation of the clinical application of CAR‐NK cells is the short persistence of CAR‐NK cells after infusion.^117^ In order to achieve cytokine‐autonomous persistence in NK cells, FT596, iPSC‐derived off‐the‐shelf CD19 CAR NK cell was engineered to express recombinant IL‐15 and IL‐15 receptor alpha (IL‐15RF). Enforced expression of IL‐15 and IL‐15RF promoted CAR NK cells expansion and persistence, and enhanced cytotoxicity against lymphoma cells.^118^

A phase I clinical trial was conducted using FT596 as a monotherapy or in combination with rituximab to treat patients with diffuse large B‐cell lymphoma. Results showed that seven out of 14 patients (50%) achieved complete remission, and 10 out of 14 patients (71%) achieved an objective response. Furthermore, the patient showed no neurotoxicity, GVHD, or other adverse effects related to FT596 treatment except for CRS, which was resolved later.^119^

Another approach to prevent the exhaustion of NK cells is to generate memory‐like phenotype immune cells, which exhibit improved persistence as well as a rapid and effective response to tumor antigens.^120^ Cytokine‐induced memory‐like (CIML) NK cells are differentiated from NK cells via coculturing with IL‐12, IL‐15, and IL‐18 in vitro, which exhibits prolonged persistence and cytotoxicity against tumor cells in preclinical and clinical trials.^121^, ^122^, ^123^, ^124^ NPM1c CAR CIML NK cells coexpressing IL‐15 autonomously showed enhanced persistence and antitumor activity NPM1 mutated AML cells in xenografts.^125^

### 5.3 Antigen heterogeneity and antigen escape

Generally, tumor‐associated antigens (TAAs) for CARs designing are highly expressed on the surface of tumor cells. However, most TAAs are expressed in tumors and normal cells, which makes it possible that normal cells will be attacked by CAR NK cells.^12^ Moreover, the expression of TAAs is significantly variable among cells in the same tumor and sometimes is downregulated to evade immune surveillance.^41^ Approaches have been used to enhance the specificity and efficiency of CAR NK cells, including the coexpression of two CAR structures in the same NK cell, and the bispecific CARs containing two target antigen recognition domains.^126^ EGFR and its mutant form, EGFRvIII, are typically overexpressed in glioblastoma (GBM) cells.^127^ Moreover, monotherapy targeting EGFR leads to the expansion of clones with EGFRvIII, and vice versa.^128^ Therefore, CAR NK‐92 cells that express scFv binding to the epitope common to both antigens were generated. Compared with the CAR NK cells singly targeting EGFR or EGFRvIII, the dual‐specific CAR NK cells showed a superior antitumor effect in GBM xenograft mice, demonstrating that dual‐targeted CAR cell therapy is a promising strategy to reduce the risk of immune escape.^129^

### 5.4 Immunosuppressive TME

#### 5.4.1 Oxidative stress

Generally, oxidative stress is caused by the increased production of reactive oxygen species (ROS) and impaired removal by antioxidants.^130^ The proliferation of tumor cells is often accompanied by high ROS generation induced by oncogene activation.^131^ While tumor cells are adaptive to high levels of ROS, the infiltration capacity and ADCC activity of NK cells are impaired.^130^, ^132^ Peroxiredoxins (PRDX) is an antioxidant enzyme and was found to eliminate H_2_O_2_ in NK cells and therefore benefit the persistence and tolerance of NK cells to oxidative stress. Based on that, PD‐L1 CAR NK cells overexpressing PRDX1 was generated and proved to improve the survival of mice transplanted with human breast tumor, which exhibited high H_2_O_2_ concentration in the local TME.^133^

#### 5.4.2 Loss of receptor mediating ADCC

**FIGURE 4:**
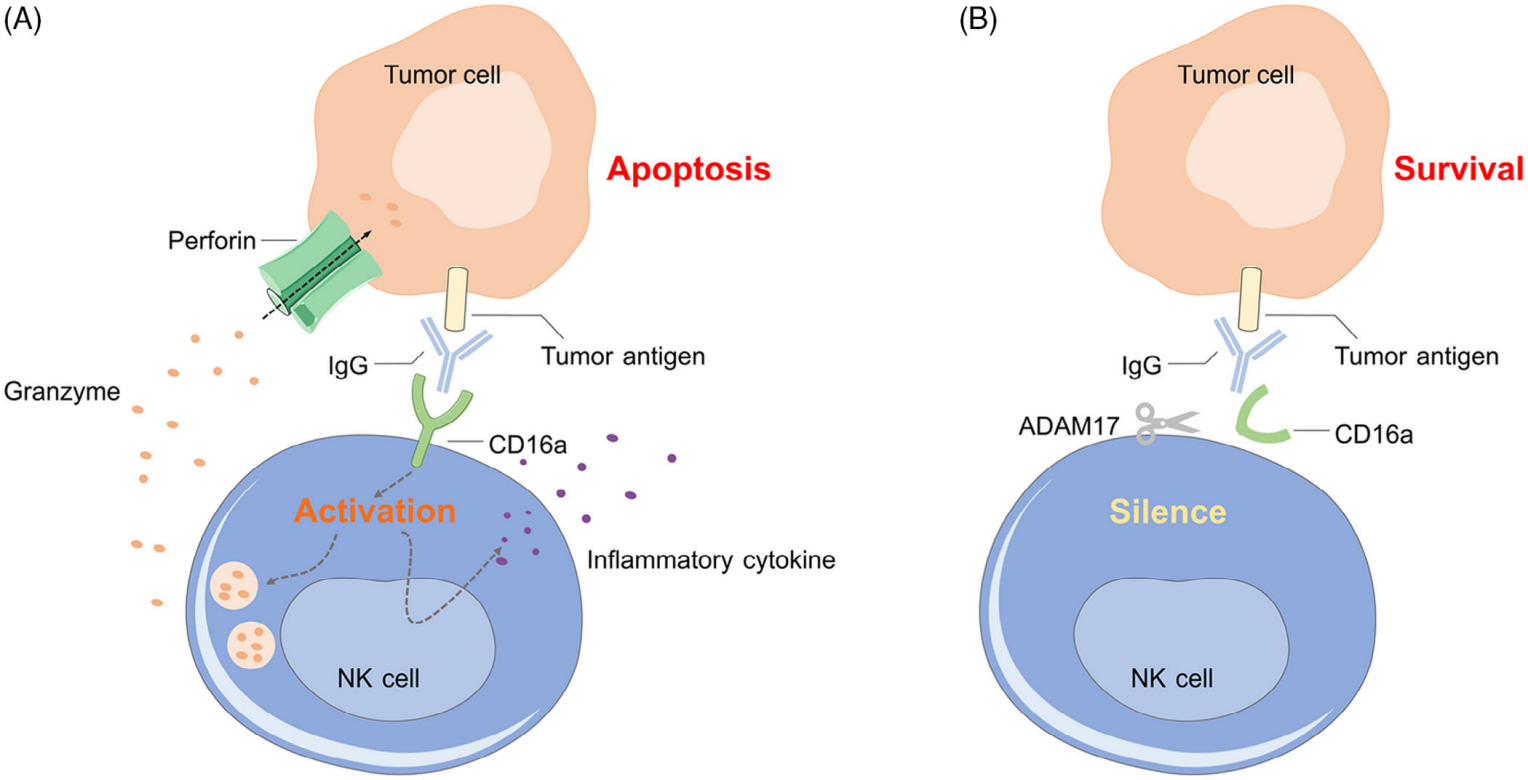
NK‐mediated ADCC. (A) CD16a, an IgG Fc receptor on the surface of NK cells, binds to IgG on target cells to induce NK ADCC, which is predominantly mediated by perforin and granzymes and leads to the apoptosis of tumor cells. (B) ADAM17 expressed by activated NK cells is involved in the protease‐driven shedding of CD16a from NK cells and thus dampens the release of effector cytokines.

CD16a is an IgG Fc receptor on the surface of NK cells, which initiates NK ADCC by binding to IgG on target cells (Figure 4A). A disintegrin and metalloproteinase 17 (ADAM17) expressed by activated NK cells is involved in the protease‐driven shedding of cell surface receptors, such as CD16a (Figure 4B).^134^ Inhibition of ADAM17 to improve the therapeutic efficacy of NK cells has been a major focus in the field.^135^ Compared with wild‐type NK cells, ADAM17 KO NK cells maintained significantly higher surface expression of CD16a after activation and displayed increased production of IFN‐γ, cytotoxicity, and ADCC activity in vitro and in vivo,^136^ implying that ADAM17 knocking out in CAR NK cells may also improve the antitumor response.

#### 5.4.3 Immunosuppressive substances

**FIGURE 5:**
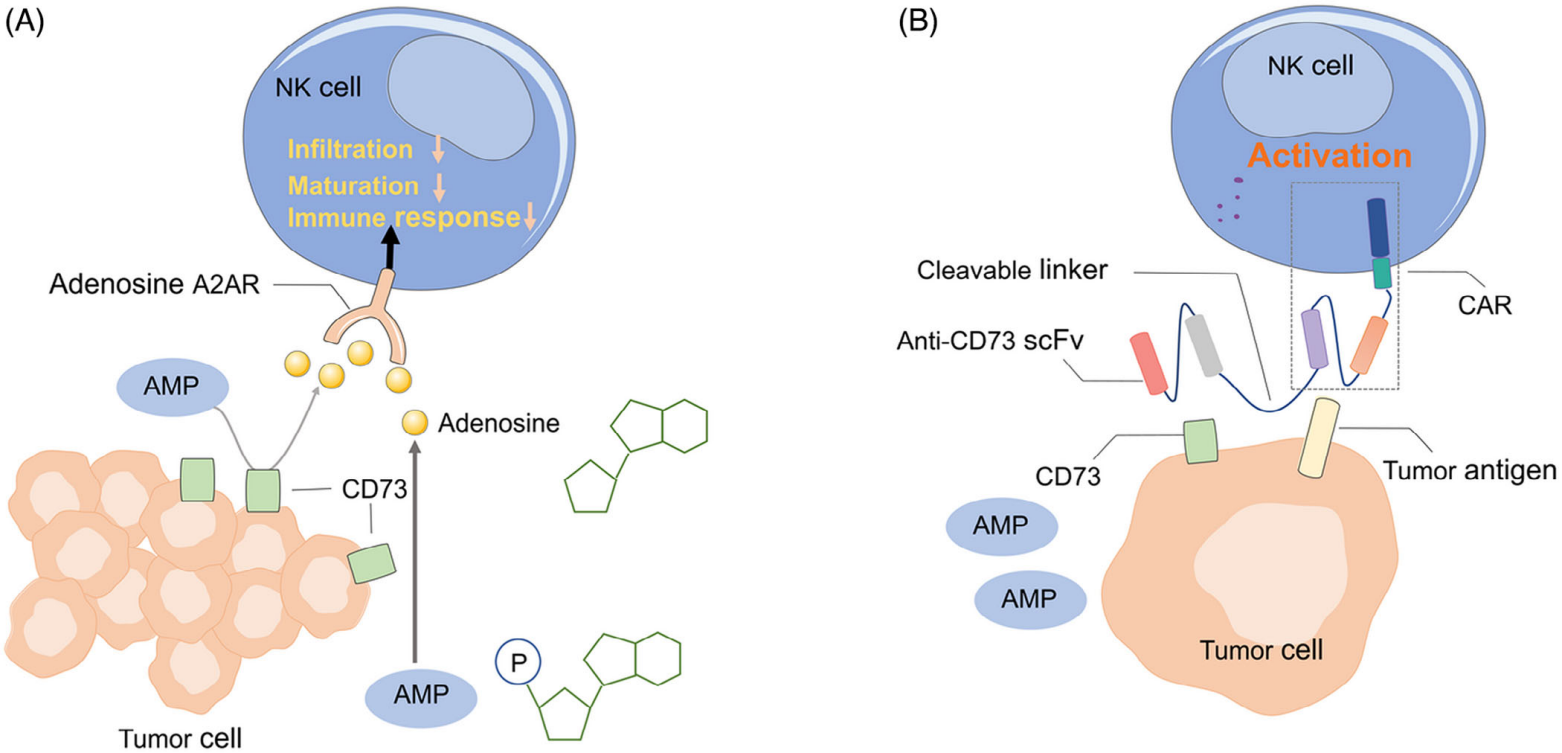
Adenosine mediates immunosuppression. (A) Adenosine is a critical immunosuppressive metabolite secreted by tumor cells, which binds the A2A adenosine receptor on NK cells to inhibit the infiltration, maturation, and immune responses of NK cells. CD73 is expressed on the tumor cell surface and converts AMP to adenosine. (B) GD2 CAR NK‐92 cells express GD2 CAR linking with an anti‐CD73 scFv via a cleavable and tumor‐sensitive linker. The anit‐CD73 antibody can be released in TME and blocks adenosine generation by CD73.

Adenosine, a critical immunosuppressive metabolite that binds to the A2A adenosine receptor (A2AR) on NK cells, was found to inhibit infiltration, maturation, and immune responses of NK cells (Figure 5A).^137^, ^138^ CD73 is an adenosine regulator in TME, which converts extracellular AMP to adenosine.^139^ High CD73 expression has been found in cancers and is associated with poor prognosis.^140^, ^141^ Previous studies showed that anti‐CD73 antibody treatment improved the infiltration of NK cells into solid tumors and significantly enhanced the therapeutic activity of immune‐checkpoint inhibitors in mice. Hence, it is worthwhile to introduce the CD73 antibody fragment into CAR NK cells. Recently, Wang et al.^142^ linked an anti‐CD73 scFv to the ganglioside GD2 CAR in NK‐92 cells via a cleavable and tumor‐sensitive linker, which specifically release an anti‐CD73 antibody into the GBM TME (Figure 5B). The blockage of CD73 promoted cytotoxicity of CAR NK cells against patient‐derived GBM cells while sparing normal cells.

In addition, TGF‐β is abundantly present in the TME and is secreted by various cell types, such as the tumor cells, regulatory T cells, and M2 macrophages, which induce inhibitory gene expression profile and suppress the cytotoxic function of NK cells.^143^ Targeting inhibition of TGF‐β via coculturing with a TGF‐β kinase inhibitor, galunisertib, or knocking out TGF‐β by CRISPR‐Cas9 prevented NK cell dysfunction as well as immune evasion by tumor cells ex vivo.^144^ What is more, the disruption of TGF‐β signaling retains the antitumor activity of NK cells in xenografts of GBM.^145^

#### 5.4.4 Immune checkpoint

Immune checkpoints, including PD‐1, CTLA‐4, CIS, and TIM‐3, are a series of molecules that prevent the activation of immune cells and inhibit immune response.^146^ The high level of checkpoints in the TME is demonstrated to be associated with cancer immune evasion as well as the exhaustion of local immune cells.^147^

Cytokine‐inducible SH2‐containing protein (CIS), a member of the suppressor of cytokine signaling family proteins, is a potent immune checkpoint in NK cells By interacting with the tyrosine kinase JAK1, CIS suppresses IL‐15 activating signaling in NK cells and inhibits the expansion and activation of NK cell.^148^ Daher et al.^90^ generated CIS‐deleted CB‐derived CD19 CAR NK cells and found CIS KO CAR NK cells showed more significant cytotoxicity against Ramos lymphoma cells and primary chronic lymphocytic leukemia cells than CAR NK cells with wild‐type CIS. PD‐1 is an inhibitory receptor expressed in T cells, which reacts with PDL1 expressed by tumor cells and prevents T cells from activating and infiltrating into tumor sites.^149^ A PD‐1 CD28 CAR structure consisting of a PD1 extracellular domain recognizing PDL‐1 expressed on tumor cells was established along with a TM, and a cytoplasmic domain of CD28 transducing activation signaling, which transduces an activating signal after reacting with PDL‐1 on the tumor cell. T cells engineered with this PD1 CD28 CAR could lead to significant regression in tumor volume because of superior T cell infiltration.^150^ Guo et al.^151^ designed a similar CAR optimized for NK cells, which contained a PD1 extracellular domain to target PDL‐1, an NKG2D hinge region, and a 4‐1BB costimulatory domain, showing an increased and stable expression on the surface of NK‐92 cells and a more remarkable antitumor efficacy against target cells.

#### 5.4.5 Migration and infiltration of NK cells into tumor sites

Rare tumor infiltration of CAR NK cells caused by inhibitory KIRs, immunosuppressive cytokines, and anatomical barriers is related to the poor prognosis.^152^ Genetic manipulation of CAR NK cells to express chemokine receptor CXCR1, which binds to the proinflammatory chemokine IL‐8 secreted by cancer cells and stroma cells in TME, enhanced CAR NK infiltration into tumor sites, reduced tumor burden and prolonged survival of tumor xenografted mice.^153^, ^154^ Similarly, enforced expression of CXCR4 in EGFR CAR NK cells also enhanced the homing capacity of CAR NK cells and increased the overall survival of mice models.^155^

Moreover, the application of TKI was found to inhibit the suppression effect of myeloid‐derived suppressor cells and regulatory T cells and increase the infiltration of lymphocytes in tumors.^156^ For example, when cotreated with regorafenib, a sorafenib‐related compound, EpCAM‐CAR‐NK‐92 cells showed increased infiltration capacity in tumor tissue therapeutic efficacy.^157^

Other approaches such as intraperitoneal injection and ultrasound‐mediated injection have been developed to directly deliver immune effector cells into tumor sites and therefore overcome the anatomical barriers. For example, MRI‐guided focused ultrasound and microbubble ultrasound contrast agents, which increased the permeability of the cerebrovasculature in target regions, were used to deliver HER2 CAR NK‐92 cells to eradicate metastatic tumors in the brain. An appreciable effect was observed as evidenced by delayed tumor progression.^158^

## 6 POTENTIAL TARGETS FOR CAR NK THERAPY

GD2 is a ganglioside highly expressed in neuroblastoma.^159^ Irradiated nonproliferating NK‐92 cells transduced with GD2 CAR were demonstrated to eliminate drug‐resistant neuroblastoma cells in vitro, and delayed tumor growth and prolonged survival were shown in xenograft mouse models treated with GD2 CAR NK‐92 cells.^20^

GPC3 is a cell surface protein highly expressed in hepatocellular carcinoma (HCC) but barely expressed in normal liver tissue.^160^ Increased expression of GPC3 is closely related to the progression of HCC.^161^ GPC3 CAR NK‐92 cells effectively reduced the tumor burden in xenografts, importantly, the degree of tumor growth inhibition was not affected by the expression level of GPC3 in HCC cells.^162^ Moreover, high levels of soluble programmed death‐ligand 1 (sPD‐L1) and membrane programmed death‐ligand 1 (mPD‐L1) impaired the antitumor capacity of CAR‐NK cells targeting GPC3. L3C7c‐Fc is a variant of sPD‐L1, which binds PD‐1 on NK cells with much higher affinity than sPD‐L1 and did not exhibit any inhibitory effect at high concentrations. The application of L3C7c‐Fc reversed the inhibition mediated by sPD‐L1, and the combination with the CAR NK 92 cell constructed with an affinity‐enhanced antibody targeting GPC3 achieved improved killing capacity toward HCC cells in the presence of sPD‐L1 or mPD‐L1 expressing tumor cells.^163^

The prostate‐specific membrane antigen (PSMA) has been a popular immunotherapy target for prostate cancer these past few years. Several clinical trials using PSMA monoclonal antibodies have been conducted in prostate cancer but the responses were observed in a minor fraction of patients.^164^, ^165^ Preclinical data showed PSMA CAR NK92 cells exhibit remarkable cytotoxicity against castration‐resistant prostate cancer cells via inducing ferroptosis, and this effect can be augmented synergistically by RSL‐3, which drives ferroptosis through GPX4 inhibition and ROS production.^166^ However, abundant IFN‐γ secreted by activated CAR NK‐92 cells was demonstrated to generate a polarized immune response and contribute to adaptive immune resistance via promoting PD‐L1 expression on tumor cells in the TME. Block the PD1–PDL1 axis via monoclonal antibody accompanied with PSMA CAR NK‐92 cell infusing had been approved to overcome this immunosuppressive effect and enhance the antitumor efficacy of CAR NK cells.^167^

NKG2D is an activating receptor of NK cells, whose membrane‐anchored ligands such as MHC class I‐related molecules MICA and MICB are overexpressed in several tumor types.^168^ In the treatment of multiple malignancies, the efficacy and safety of NKG2D‐expressing CAR T cells have been proven through previous clinical trials.^169^, ^170^ Nevertheless, the downregulation of NKG2DLs on tumor cells and the upregulated levels of soluble NKG2DLs in the serum of patients, which induce NKG2D internalization and degradation, impaired the efficacy of NKG2D expressing immune cells.^171^, ^172^ Recently, a CAR with two NKG2D structures and two ErbB2 scFv domains was designed to address the interference by soluble NKG2DLs and redirect NK cells to ErbB2‐positive tumor cells. Bispecific CAR NK cell was exempt from inhibition of soluble MICA and exhibited significantly enhanced antitumor activity in the mouse GBM model.^173^

As a TM glycoprotein highly expressed in myeloma cells, B cell maturation antigen (BCMA) is the leading target for CAR NK therapy for multiple myeloma (MM).^174^ BCMA CAR NK cells coexpressing cytokine receptor CXCR4, which facilitates trafficking of NK cells to BM via binding to CXCR12 expressed by BM cells, exhibited improved migration and BM homing capacity.^175^

## 7 CLINICAL TRIALS FOR CAR NK THERAPY

In recent years, numerous preclinical studies demonstrated the therapeutic potential of CAR NK cells in treating hematological malignancies and solid tumors, and many CAR NK clinical trials are ongoing (Table 3). Two trials demonstrating evidence of clinical success have been completed although many other clinical trials are still ongoing or recruiting.

**TABLE 3:** Clinical trials of CAR NK cell therapy.

Tumor type	Target	Phase	Source	NCT number
ALL	CD19	I	NA	NCT05563545
MM	BCMA	Early phase I	NA	NCT05652530
B‐cell NHL	CD19	I	NA	NCT04887012
AML	CD123	Early phase I	NA	NCT05574608
ALL/CLL/NHL	CD19	I	NA	NCT05410041
Ovarian cancer	NKG2D	NA	NA	NCT05776355
AML	CD33/CLL1	Early phase I	NA	NCT05215015
AML	NKG2D	NA	NA	NCT05734898
ALL/CLL/NHL	CD19	I	UCB	NCT04796675
Refractory metastatic colorectal cancer	NKG2D	I	NA	NCT05213195
Diffuse large B cell lymphoma	CD19	Early phase I	NA	NCT05673447
Ovarian cancer/testis cancer	Claudin6	I/II	PB	NCT05410717
Advanced solid tumors	5T4	Early phase I	NA	NCT05194709
B‐cell NHL	CD19	I	UCB	NCT05472558
NHL	CD19	Early phase I	NA	NCT04639739
ALL/CLL/NHL	CD19	Early phase I	NA	NCT05739227
Solid tumors	NKG2D	I	Autologous or allogeneic	NCT03415100
Leukemia/myeloid	CD33	I	NA	NCT05008575
MM	BCMA	Early phase I	UCB	NCT05008536
B cell lymphoma	CD19	I/II	NA	NCT05570188
Small cell lung cancer	DLL3	I	NA	NCT05507593
Refractory B‐cell lymphoma	CD22	Early phase I	NA	NCT03692767
Refractory B‐cell lymphoma	CD19	Early phase I	NA	NCT03690310
Solid tumor	ROBO1	I/II	NA	NCT03940820
AML	CD33/CLL1	I	NA	NCT05987696
Breast cancer	SZ011	Early phase I	NA	NCT05686720
Hepatocellular carcinoma	SZ003	NA	NA	NCT05845502
ALL/CLL/NHL	CD33	I/II	NA	NCT02944162
MM	BCMA	I/II	NA	NCT03940833
Ovarian epithelial carcinoma	SZ011	Early phase I	NA	NCT05856643
Pancreatic cancer	ROBO1	I/II	NA	NCT03941457
Refractory B‐cell lymphoma	CD19/CD22	Early phase I	NA	NCT03824964
B‐cell lymphoblastic leukemia/lymphoma	CD19	I/II	NA	NCT05654038
Refractory metastatic colorectal cancer	NKG2D	Early phase I	NA	NCT05248048
Gastroesophageal	PD‐L1	II	NA	NCT04847466
Pancreatic cancer/ovarian cancer/adenocarcinoma	TROP2	I/II	UCB	NCT05922930
T‐lymphoblastic lymphoma/acute lymphocytic leukemia	CD7	I	NA	NCT04004637
B Cell NHL	CD19/CD70	I	UCB	NCT05667155
ALL/CLL/NHL	CD19	I/II	NA	NCT02892695
Hematological malignancy	CD5	I/II	UCB	NCT05110742
B cell lymphoma/MDS/AML	CD70	I/II	UCB	NCT05092451
AML/MDS	NKX101	I	NA	NCT04623944
B Cell NHL	CD19/CD70	I/II	UCB	NCT05842707
T‐cell leukemia/T‐cell lymphoma	CD7	Early phase I	NA	NCT04264078
T‐cell ALL/AML/NK cell lymphoma	CD7	I/II	NA	NCT04033302
ALL/CLL/B‐cell lymphoma	CD19	I	NA	NCT04796688
Locally advanced or metastatic solid tumors	5T4	Early phase I	NA	NCT05137275
Lymphomas	CD30	I/II	NA	NCT02274584
Hematologic malignancies	RD13‐01	I	NA	NCT04538599
Relapsed/refractory MM	LUCAR‐B68	I	NA	NCT05498545
Relapsed or refractory B Cell NHL	TAK‐007	II	NA	NCT05020015
T‐ALL/T‐cell lymphoma	SENL101	I	NA	NCT05398614
Advanced pancreatic carcinoma	CD276	I/II	NA	NCT05143151
MM	FT576	I	NA	NCT05182073
AML	QN‐023a	I	NA	NCT05665075
AML	QN‐023a	I	NA	NCT05601466
Epithelial ovarian cancer	Mesothelin	Early phase I	NA	NCT03692637
Solid tumor	RD133	Early phase I	NA	NCT05166070
Relapsed/refractory MM	ALLO‐715	I	NA	NCT04093596
Solid tumor	RD133	Early phase I	NA	NCT05141253
Relapsed/refractory large B cell lymphoma	ALLO‐647	II	NA	NCT05714345
Hematopoietic/lymphoid cancer	PEPRR	III	NA	NCT00833898
EBV‐associated lymphomas	KSD‐101	Early phase I	NA	NCT05882305
EBV‐associated hematologic neoplasms	KSD‐101	I	NA	NCT05635591
NHL	CD19	Early phase I	NA	NCT03910842
Blastic plasmacytoid dendritic cell neoplasm (BPDCN)	UCART123	I	NA	NCT03203369
Indolent NHL/aggressive NHL	CNTY‐101	I	NA	NCT05336409
Refractory/relapsed NHL	CD19	I	NA	NCT05618925

Abbreviations: ALL, acute lymphocytic leukemia; CLL, chronic lymphocytic leukemia; MDS, myelodysplastic syndrome; MM, multiple myeloma; NA, not available.; NHL, non‐Hodgkin lymphoma.

*Data sources*: ClinicalTrials.gov.

The clinical success of CD19 CAR T cells in the treatment of malignant hematological diseases attracted more attention to applying CD19 CAR to NK cells.^176^, ^177^ The use of anti‐CD20 mAbs such as rituximab and obinutuzumab has dramatically improved the outcome of B‐cell non‐Hodgkin lymphomas (bNHL).^178^, ^179^ However, relapse and the development of refractory disease are common among bNHL patients treated with CD20 mAbs because of the lack of CD20 expression of tumor cells and deficiencies of host immune factors.^180^ CD19 CAR NK cell exhibited significantly superior cytotoxicity against anti‐CD20 resistant lymphoma cells via longer contact duration with target cells, increased secretion of FasL, C‐C motif chemokine ligand (CCL3, and IL10 as well as inducing genes related to mTOR and cell cycle checkpoint activation.^181^ Moreover, PB‐derived CD19 CAR NK cells infusion combined with anti‐CD20 mAb treatment increased the release of effector cytokines and tumor cell clearance in mice xenografted with B‐ALL cells while retaining the expression and function of native activating receptors.^182^ IL‐15‐expressed CD19 CAR NK cells have been generated and evaluated in a clinical trial for the treatment of patients with relapsed or refractory CD19‐positive CLL or NHL. Seven out of 11 patients exhibited a complete response without symptoms of CRS, neurotoxicity, or GVHD despite the HLA mismatch between the patients and CAR‐NK cells.^18^ These encouraging results shed light on the further development of off‐the‐shelf CAR‐NK cell products in clinical application.

Based on the promising preclinical results NK cells expressing NKG2D CARs with the CD3zeta or DAP12 intracellular signaling domain showed significant immune responses, slower disease progression, and prolonged median survival time in mice with human colorectal cancer xenografts. Xiao et al.^109^ conducted a clinical trial study of NKG2D CAR NK cells in three patients with chemotherapy‐refractory metastatic colorectal cancer. Two patients were intraperitoneally injected with low‐dose CAR NK cells and a reduction of ascites as well as ascites tumor cells was observed. Rapid tumor regression in the liver region was observed in the third patient with liver metastasis after treating with the CAR‐NK cells. Besides grade 1 CRS, no serious adverse effects were observed in any of the three patients. GVHD did not develop in the two patients treated with haploidentical NK cells.^109^ Many problems regarding the safety and efficacy of CAR‐NK cell therapy still need to be solved before CAR NK becomes a routine therapy.

## 8 ALTERNATIVE IMMUNE CELLS FOR CAR EXPRESSION

Apart from NK cells and T cells, other immune cells, including invariant NK T (iNKT) cells, γδT cells, macrophages, dendritic cells, and neutrophils possessing specific advantages have been explored as candidates for CAR‐engineering cells, and some CAR‐based products using these cells are being tested in clinical trials.

### 8.1 iNKT cells

iNKT cells are a small population of αβ T lymphocytes expressing a semi‐invariant T cell receptor, which recognizes lipids presented by CD1d, a nonpolymorphic MHC class I‐like molecule. Similar to innate immune cells such as NK cells, iNKT cells are stimulated by a variety of cytokines, such as IL‐12, IL‐18, and IL‐23 apart from TCR stimulation.^183^ Once recognizing the lipids presented by CD1d or infiltrated into tumor sites with chemokines such as CCL2 and CCL20, iNKT cells are activated and exhibit antitumor activity through direct lysis of target cells or releasing of cytokines, which recruit other immune cells to the TME and potentiated tumor‐specific immunity. Besides, adoptive iNKT cells will not cause GVHD due to a lack of MHC expression.^184^ Additionally, iNKT cells exhibit strong effector function and self‐renewing capacity.^185^ These merits make iNKT cells promising candidates for CAR‐based immunotherapy.

CAR iNKT cells targeting tumor antigens such as CD19, CD38, BCMA, CSPG4, and GD2 have been generated, and the efficacy has been evaluated in preclinical models or clinical trials.^186^, ^187^, ^188^, ^189^ Moreover, the CAR19‐iNKT cells were found to eradicate the lymphomas in the brain, and in combination with all‐trans retinoic acid, which enhanced CD1D expression on tumor cells, the cytotoxicity of CAR19‐iNKT cells was further enhanced. However, the potential toxicity of CAR‐iNKT cells, especially the “off‐tumor, on‐target” effect of CD1d‐expressing cells still needs to be determined.^190^ In another study, iNKT cell expression GD2 CAR exhibited better antitumor efficacy than GD2 CAR T cells in neuroblastoma‐bearing mice. Additionally, the antitumor activity of GD2 CAR iNKT cells could further be strengthened when IL‐15 was coexpressed in the effector cells. Therefore, a clinical trial was conducted to evaluate the antitumor effect and safety of GD2 CAR iNKT cell coexpressing IL‐15 in neuroblastoma (NCT03294954).^191^ The results showed that GD2 CAR‐IL15 NKT therapy was safe and could mediate objective responses in patients with neuroblastoma. One of two patients achieved tumor regression with no symptom of toxicity.^192^

### 8.2 γδT cells

γδT cells account for 1−5% of all the circulating T cells but represent a major part (10–100%) of epithelial tissue. Epithelial γδT cells play a vital role in mediating a rapid immune defense response against invading pathogens while maintaining immune tolerance to microscopic organisms.^193^ γδT cells can be activated via several surface receptor‐ligand signaling pathways instead of class MHC‐dependent fashion.^194^ Upon activation, γδT cells directly kill target cells by secreting cytotoxic molecules like perforin and granzymes or expressing apoptosis‐inducing ligands including TRAIL and FasL.^195^ Similar to NK cells, γδT cells express CD16 on the surface and therefore mediate ADCC via binding to the Fc region of antibodies targeting tumor cells.^196^ By releasing cytokines, including GM‐CSF, IFN‐γand TNF, γδT cells can recruit and trigger other immunity response cells like αβ cells and dendritic cells to kill target cells.^194^ Moreover, γδT cells recognize target cells in a manner that is not dependent on MHC molecules and therefore do not cause GvHD, making them an ideal source for CAR engineering. However, secretion of IL‐17 endows γδT cells capacity to promote the proliferation and migration of tumor cells and VEGF‐dependent angiogenesis mainly by inhibiting the function of CD8 T cells.^197^

γ9δ2T cells, a γδT cell subset, were found to be relatively abundant in the PB and can be expanded to a large number for clinical use ex vivo. Preclinical results showed that CAR γδT cells exhibited potent antitumor activity in vitro and in vivo. Expression of GD2 CAR γδT cells retained the functions of antigen cross‐presentation to αβT cells and migration toward tumor cells while exhibiting enhanced tumor‐killing capacity.^198^ In another study, γδT cells separated from PBMC were transduced with a CD19 CAR and expanded further using K562‐based feeder cells and showed an improved antitumor activity in the CD19 B‐cell leukemia mice model.^199^ Several clinical trials of CAR γδT cells are anticipated, which are going to evaluate the safety and efficacy in patients with hematopoietic malignancies (NCT05388305, NCT05388305, NCT02656147, NCT04702841).

## 9 CONCLUSIONS

As an innovative and effective cellular immunotherapy, CAR NK cell owns several advantages compared with CAR T cell therapy. Owing to the independence of MHC‐matching and abundant sources, CAR NK cells can be available as off‐the‐shelf products, reducing the cost and waiting time for immediate clinical use. Relative shorter lifespan and alternative cytokine secretion spectrum of CAR NK cells reduce the risk of “on‐target, off‐tumor” toxicity, and CRS. Preclinical studies have shown the great promise of CAR NK cells in a wide range of tumor types. Besides, the safety as well as tolerance of CAR NK cell therapy have been shown in clinical trials.

Although tremendous progress has been achieved, and CAR NK cell adoptive immunotherapy is being tested in multiple clinical trials and offers the possibility of a safer, off‐the‐shelf, antitumor therapy, the clinical translation of CAR NK cell therapy is still in the early stage compared with CAR T cell therapy. Some potential issues present in CAR NK treatment remain open, including tailor‐made CAR structure for NK cells, appropriate transduction system for CAR gene expression in NK cells, especially to naïve NK cells, and optimal source and expansion conditions for CAR NK cell manufacture. Besides, immunosuppressive TME that impairs NK cell activation, persistence, and infiltration is another obstacle to CAR NK therapy that needs to be solved.

Reprogramming CAR‐NK cells via gene editing or including novel genes in CAR structure was proved to significantly improve the efficacy of CAR NK cells. Additionally, the combination therapy strategies, such as the integration of mAbs and small‐molecule inhibitors to block the checkpoint and deplete the immunosuppressive metabolites, provide a coordinated effect and increase the resistance of CAR NK cells to the TME.

With the development of CAR NK cell therapy in clinical practice, a number of ethical issues have been raised and must be solved. Strict standards to monitor and evaluate the CAR NK cell manufacturers and medical institutions should be formulated to standardize the techniques of enriching NK cells and ensure their safety and effectiveness during treatment. Patients must be fully informed about the potential benefits and risks of CAR NK cell therapy. Additionally, NK cells for immunotherapy are obtained from autologous or allogeneic sources, allowing for the “off‐the‐shelf” availability of CAR NK cells, which can be immediately used for patients at lower cost. On the other hand, ensuring fair and equitable allocation of limited sources should be considered to avoid exacerbating existing health disparities.^200^

In conclusion, with the increasing evidence of safety and efficacy in clinical trials, and new strategies to address the remaining challenges, it is believed that immunotherapies based on CAR NK cells will continue to develop and represent a bright future for the treatment of hematological malignancies and solid tumors.

## AUTHOR CONTRIBUTION

*Writing—original draft preparation*: Y. W. *Writing—review and editing*: J. S., Y. W., S. J. J., Q. Q. Z., N. L., R. Y. C., and S. A. A. *Visualization*: Y. W. *Supervision*: J. J. and J. S. All authors have read and agreed to the published version of the manuscript.

## CONFLICT OF INTEREST STATEMENT

The authors declare no conflict of interest.

## ETHICS STATEMENT

Not applicable.

## Data Availability

Not applicable.
